# Bilateral Thoracic Outlet Syndrome from Anomalous 8th Cervical Vertebrae Ribs

**DOI:** 10.1055/s-0042-1753541

**Published:** 2022-07-18

**Authors:** Scott Ferris, Sarah Lonie

**Affiliations:** 1 Department of Plastic, Hand and Faciomaxillary Surgery, Alfred Health, Melbourne, Victoria, Australia

**Keywords:** thoracic outlet syndrome, eighth cervical vertebra, cervical rib, brachial plexus

## Abstract

Thoracic outlet syndrome (TOS) is a group of diverse disorders resulting from compression of neurovascular structures as they pass from the lower neck to upper limb. Neurological symptoms, such as pain, weakness, or paraesthesia, are much more common than vascular symptoms such as pallor or venous congestion. Anatomical abnormalities can contribute to this condition. Thirty percent of patients with TOS can have a cervical rib, arising from the transverse process of the 7th cervical vertebra, compared with 1% of the general population. We report the first case in the literature of neurogenic TOS from a cervical rib arising from a supernumerary 8th cervical vertebra. This patient had immediate improvement in TOS symptoms following scalene muscle surgery and resection of cervical and first thoracic ribs.

## Background

Thoracic outlet syndrome (TOS) is caused by compression of neurovascular structures passing through the cervicoaxillary canal. Symptoms can include pain, paraesthesia, weakness, coldness, pallor, or venous congestion depending on the compromised structure. Neurological symptoms (95%) are much more common than vascular symptoms (5%). 1 Neurological and vascular symptoms can certainly coexist and often have overlapping presentations. Compression levels are commonly divided into three areas: the interscalene triangle between anterior, middle scalenes, and first rib, the costoclavicular space between clavicle and first rib, and the subcoracoid space inferior to pectoralis minor. 2 As a dynamic space, compression of the neurovascular bundle and symptoms are often worse with use of the upper limb.

A range of anatomical anomalies have been reported associated with TOS. 3 4 Cervical ribs are usually an incidental finding on chest X-ray, arising from the transverse process of the 7th cervical vertebra. They exist in 1% of the general population, with most patients being asymptomatic and 10% manifesting compressive symptoms. 5 Of patients with TOS, a cervical rib has been reported to be present in almost 30%. 2 6

We report the first case in the literature of neurogenic TOS from a cervical rib arising from an anomalous supernumerary 8th cervical vertebra.

## Case Report

A 27-year-old female presented with symptoms of bilateral neurogenic TOS. She was otherwise well but described 5 years of symptoms with upper limb numbness particularly in the C8/T1 dermatomal distribution, and pain in both shoulders, neck, and scapulae. All symptoms were worse with activity and limb elevation. She had no obvious precipitating trauma. As a result of her symptoms she was unable to work.

On examination she had bilateral flattened thenar eminences and reduced power in her first dorsal interosseous and abductor digiti minimi muscles. She failed the elevated arm stress test bilaterally, right earlier than left sides, and lost the right radial pulse at 90 degrees shoulder abduction with 50 degrees external rotation and the left radial pulse at 90 degrees shoulder abduction with 90 degrees external rotation.

**Fig. 1:**
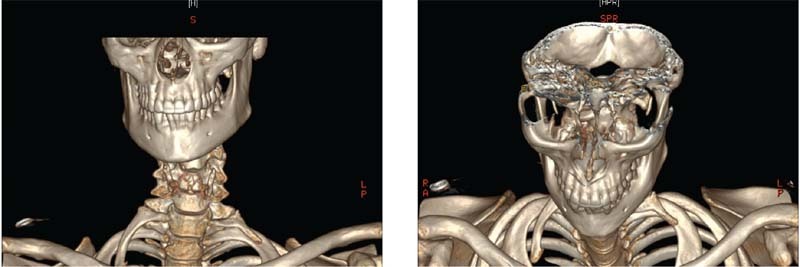
( **A** ) Cervical rib with subclavian artery and middle and inferior trunks overlying following anterior scalenotomy. ( **B** ) Blue dash line indicating insertion of anterior scalene to cervical rib.

She underwent neurophysiology tests and imaging including magnetic resonance imaging of the brachial plexus and cervical spine X-rays and computed tomography. All were unremarkable except for the finding of a supernumerary C8 vertebra with bilateral cervical ribs arising from this C8, with the right larger than left ( Fig. 1A, B ).

In terms of patient-reported measures, she scored 42/93 in Brachial Assessment Tool and had a QuickDASH score of 61.36. She was consented and scheduled to undergo staged thoracic outlet decompression, starting with the more severe right side.

**Fig. 2:**
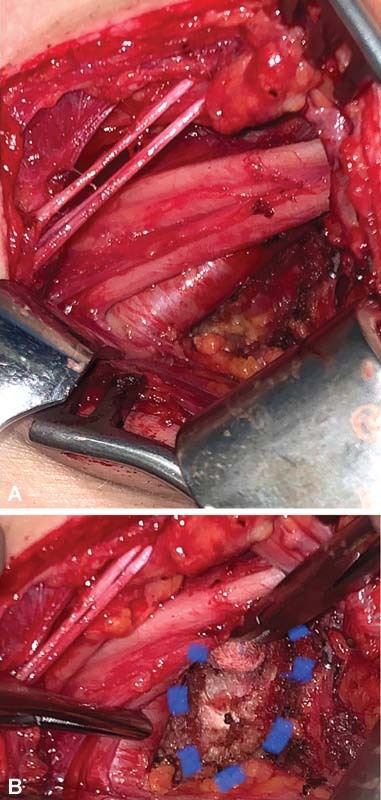
Cervical spine computed tomography (CT) demonstrating C8 vertebrae with bilateral ribs, right larger than left. ( **A** ) Anterior view. ( **B** ) Superior view.

**Fig. 3:**
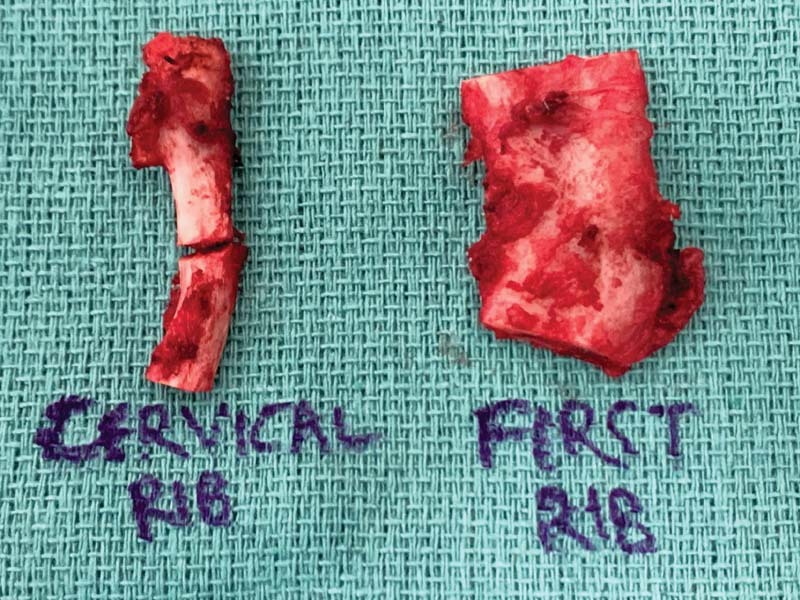
Portions of resected cervical rib and first rib as labeled.

The patient underwent release of the right thoracic outlet with multiple neurolyses, scalenotomies, and resection of both the anomalous C8 cervical rib as well as resection of the first thoracic rib segment subjacent to the clavicle. This was done via a supraclavicular approach ( Fig. 2A, B ). Intraoperatively, a long upper trunk of the brachial plexus was found with an atypical contribution to the phrenic nerve from its anterior surface. The anterior scalene was inserting into the cervical rib, compressing the subclavian artery and middle and inferior trunks of the brachial plexus. These neurovascular structures were running tautly over the cervical rib. Fibrinous scar in the region was consistent with long-standing local inflammation. Once an anterior scalenotomy had been performed releasing its insertion, the anterior and middle portions of cervical rib were removed. On shoulder motion with abduction and external rotation, there was ongoing costoclavicular impingement. The scalenus medius insertion was therefore released off the first rib and the relevant portion of this rib was segmentally resected to decompress the costoclavicular impingement ( Fig. 3 ). The patient had immediate and sustained postoperative improvement in symptoms with arm activities including high elevation of the limb.

## Discussion

Embryologically, cervical vertebrae formation is controlled by Hox genes and seven cervical vertebrae is remarkably consistent across all mammals. 7 This consistency is proposed to be due to Hox genes also being responsible for neural defects, neonatal malignancies, or stillbirth. A range of mild anomalies of the cervical spine exist, commonly fusion as seen in Klippel-Feil syndrome and cervical ribs. 8 9 There is only one other report of an eighth cervical vertebra 10 in the literature. This was in an 11-year-old boy, with an incidental finding of 8 cervical and 13 thoracic vertebrae, when X-rays were performed due to neck trauma. There were no compressive thoracic outlet symptoms reported in this case.

Gruber classified the cervical rib anomaly in 1869 into four types. 5 The cervical rib in type 1 extends just beyond the transverse process of C7; in type 2 beyond the transverse process but not connected to the first rib; in type 3 the cervical rib is partially fused through fibrous bands or cartilage to the first rib; and in type 4 the cervical rib is united with the first rib. Cervical ribs are positioned such that they narrow the interscalene interval through which the subclavian artery and trunks of the brachial plexus are transmitted from neck to axilla. The mere presence of cervical ribs certainly does not mandate removal. Despite the existence of the anatomical variation, more than the bony anomaly alone is commonly required to cause symptoms. In 80% of patients a supervening trauma will bring on TOS symptoms where none existed prior to the trauma. 11 Spontaneous symptoms (with no known trauma or other precipitant) are however more likely in complete, type 4 cervical ribs with 50% incidence of spontaneous symptoms compared with a 20% incidence with incomplete ribs. In our patient, with incomplete cervical ribs, the spontaneous and severe symptoms could be attributed to traction and compression of the neurovascular structures across the cervical rib with an additional cervical vertebra.

First-line treatment for TOS is nonsurgical with posture modification, exercises, and strengthening. After a period of 3 to 12 months, failing sufficient symptom improvement, surgical management is considered. In the presence of cervical ribs requiring operative management for TOS, Chang et al and Sanders and Hammond now advocate for the removal of cervical and first ribs to avoid need for revisionary surgery, without increased morbidity. 5 11 Sanders and Hammond report 24% failure of TOS surgery on cervical ribs including first rib removal but 41% failure without first rib removal. 11

## Conclusion

We report the first case of a supernumerary 8th cervical vertebra with its anomalous cervical rib being associated with TOS.
